# Novel targetable biomarkers in clear cell carcinoma of the breast uncovered by molecular profiling: A study of nine cases

**DOI:** 10.1111/tbj.13842

**Published:** 2020-04-11

**Authors:** Faruk Skenderi, Juan Palazzo, Jeffrey Swensen, Rebecca Feldman, Elma Contreras, Elena Florento, Zoran Gatalica, Semir Vranic

**Affiliations:** ^1^ Department of Pathology Clinical Center University of Sarajevo Sarajevo Bosnia and Herzegovina; ^2^ Baptist Hospital of Miami Miami FL USA; ^3^ Caris Life Sciences Phoenix AZ USA; ^4^ Creighton University School of Medicine Phoenix AZ USA; ^5^ College of Medicine QU Health Qatar University Doha Qatar

**Keywords:** breast cancer, clear cell carcinoma, immunotherapy, molecular profiling, targeted therapy

## Abstract

We profiled nine pure clear cell carcinomas of the breast using massively parallel DNA and RNA sequencing (NGS), in situ hybridization (ISH), and immunohistochemistry (IHC). All cases were primary mammary clear cell carcinomas that were diagnosed in female patients (mean age: 53.4 years; range: 31‐69 years). Based on our findings, we conclude that the majority of clear cell carcinomas are ER/PR positive and consequently amenable to anti‐ER treatment modalities. A subset of clear cell carcinomas also harbored alterations in PIK3CA/PTEN/AKT pathway, particularly PTEN, indicating a potential benefit of PI3K/Akt/mTOR inhibitors. The status of I‐O biomarkers in clear cell carcinomas indicates a limited therapeutic benefit of immune checkpoint inhibitors (against PD‐1/PD‐L1).

Clear cell differentiation may be occasionally seen in various subtypes of breast cancer, but pure forms of clear cell carcinoma (>90% clear cell morphology) are exceptionally rare. These cancers are characterized by neoplastic cells with an abundant and clear cytoplasm that typically contains glycogen.^1^ This type of cancer is considered a distinct cyto‐morphological pattern of invasive breast carcinoma of no special type (IBC‐NST).^1^ The clinical data on clear cell carcinoma are limited and predominantly include small retrospective studies that reported the conflicting results.^2^ However, a recent Surveillance, Epidemiology, and End Results (SEER) study revealed that clear cell carcinomas tend to be pathologically high‐grade cancers that clinically present at an advanced stage and have poor outcomes.^3^ Apart from reports of variable steroid receptor (ER and PR) and HER2 positivity, no studies have systematically explored molecular features and potentially targetable biomarkers in clear cell carcinomas.^2^ Herein, we profiled nine pure clear cell carcinomas of the breast using massively parallel DNA and RNA sequencing (NGS), in situ hybridization (ISH), and immunohistochemistry (IHC). All cases were primary mammary clear cell carcinomas that were diagnosed in female patients (mean age: 53.4 years; range: 31‐69 years) (Figure 1). Six out of nine cases were periodic acid‐Schiff (PAS) positive and PAS‐diastase sensitive (glycogen‐rich). The NGS platform covered exons from 592 genes (SureSelect XT, Agilent, Santa Clara, CA; and the NextSeq instrument, Illumina, San Diego, CA). The tumor mutational burden (TMB) was considered high if ≥11 mutations/megabase were detected^4^ (Table 1). Microsatellite instability (MSI) was calculated from NGS data by direct analysis of short tandem repeat tracts in the target regions of sequenced genes.^5^ The ArcherDX FusionPlex Assay (ArcherDX, Boulder, CO) was used for gene fusion assessment (n = 54; Table 1). The following biomarkers were tested by immunohistochemistry (IHC): steroid receptors (ER, PR, AR, ARv7), HER2, pTRK, PD‐L1, PTEN, and mismatch repair proteins (MLH1, MSH2, MSH6, and PMS2) (clones and thresholds for positivity are provided in Table S1).^4^, ^6^, ^7^ Both ER and PR were positive in the majority of cases (8+/9 cases each) (Table 1). AR was positive in 7/9 cases (78%) without the presence of the ARv7 splice variant. No case was HER2 positive by IHC or ISH (0%). Pathogenic mutations were detected in three cases: *PIK3R1* and *BRCA2* (#1); *TP53, PTEN*, and *CDKN2A* (#2); and *TP53* and *BCOR1* genes (#3) (Table 1). PTEN protein loss was confirmed by IHC in the one *PTEN*‐mutated case as well as in two additional cases without detectable *PTEN* gene mutations (Table 1). No gene fusion was detected in any of the cases. Low PD‐L1 expression (1%‐10%) was exclusively seen in immune cells in 3/8 cases (Figure 1); notably, one of the PD‐L1 + cases had an underlying *PTEN* gene mutation (Table 2, Figure 1). All tested cases (n = 8) were MSI stable (by NGS or IHC) and had low TMB (3‐7 mutations/megabase) (n = 4) (Table 2).

**Figure 1:**
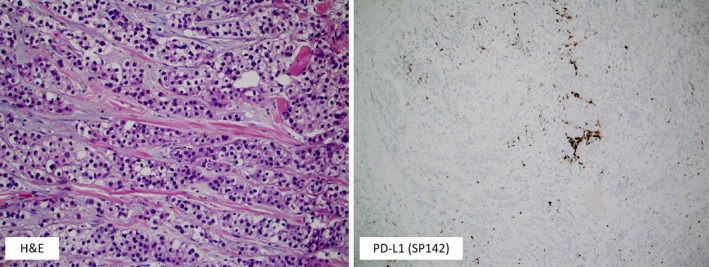
Case #4: a tumor with clear cell morphology (left image, 20x) with PD‐L1 expression (~10%) detected exclusively in immune cells (right image) (PD‐L1, SP142 clone, Ventana, 20x)

**Table 1:** Overview of the potentially targetable biomarkers in cell carcinomas of the breast

Case	ER and PR	AR and ARv7	HER2 status	Mutational profile	Gene fusions (NTRK)^b^
#1	ER (+), PR (+)	AR (+)	Negative	None	None
#2	ER (+), PR (+)	AR (−)	Negative	n/a	None
#3	ER (+), PR (−)	AR (+), ARv7 (−)	Negative	None	None
#4	ER (+), PR (+)	AR (+), ARv7 (−)	Negative	*BRCA2, PIK3R1,* PTEN loss^a^	None
#5	ER (+), PR (+)	AR (+), ARv7 (−)	Negative	n/a	None
#6	ER (+), PR (+)	AR (+), ARv7 (−)	Negative	*PTEN, TP53, CDKN2A*	None
#7	ER (+), PR (+)	AR (+), ARv7 (−)	Negative	n/a	None
#8	ER (+), PR (+)	AR (+), ARv7 (−)	Negative	n/a	None
#9	ER (−), PR (+)	AR (−)	Negative	*TP53, BCOR,* PTEN loss^a^	None

Abbreviations: AR, androgen receptor; ARv7, androgen receptor splice variant 7; ER, estrogen receptor; HER2, human epidermal growth factor receptor 2; NTRK, neurotrophic receptor tyrosine kinase; PR, progesterone receptor.

^a^ PTEN loss was observed by immunohistochemistry. Case #6 with *PTEN* gene mutation also exhibited PTEN protein loss by IHC.

^b^ ArcherDX FusionPlex Assay (ArcherDX, Boulder, CO) was used to assess gene fusions (n = 54) (the panel is available here: https://www.carismolecularintelligence.com/wp‐content/uploads/2017/03/TN0276‐v14_Profile‐Menu.pdf). NTRK status was also assessed by immunohistochemistry.¸

**Table 2:** The status of immuno‐oncology (I‐O) biomarkers in clear cell carcinomas of the breast

I‐O biomarkers	Status in clear cell carcinomas
PD‐L1 expression (n = 8)	3/8 positive in immune cells (1%‐10% positivity) No expression in cancer cells
Tumor mutational burden (TMB)^a^ (n = 4)	4/4 low (5‐7 mutations/megabase)
Microsatellite instability (MSI) (n = 8)	8/8 MSI stable

Abbreviations: I‐O, immuno‐oncology.

^a^ TMB was considered high if ≥11 mutations/megabase were detected. The estimated threshold was based on a cohort of 603 triple‐negative breast carcinomas of no special type using an 80th percentile cutoff value.^14^

Based on our findings, we conclude that the majority of clear cell carcinomas are ER/PR positive and consequently amenable to anti‐ER treatment modalities. Although not routinely assessed, the importance of AR expression in breast cancer has been increasingly recognized,^1^ particularly in triple‐negative breast cancer (TNBC). Although we found frequent AR overexpression in clear cell carcinomas without the ARv7 splice variant, potential therapeutic benefit of anti‐AR–based therapy alone in clear cell carcinomas expressing ER is uncertain. Alterations within the PI3K/Akt/mTOR pathway are among the most common genomic alterations in breast cancer.^8^ A subset of clear cell carcinomas also harbored alterations in this pathway, particularly PTEN, indicating a potential benefit of PI3K/Akt/mTOR inhibitors. A complete loss of PTEN protein expression without detected *PTEN* gene mutations in two cases indicates an alternative silencing mechanism of this important tumor suppressor. The observed alterations in clear cell carcinomas may be clinically relevant given that the Food and Drug Administration (FDA) has recently approved a PIK3CA inhibitor Piqray (alpelisib) combined with fulvestrant for the treatment of ER+/PIK3CA‐mutated metastatic breast carcinomas. In addition, two of three clear cell carcinomas with PI3K/PTEN alterations were AR+. A recent clinical trial showed the therapeutic benefit of combined anti‐AR (enzalutamide) and PIK3CA inhibitor (taselisib) in TNBC patients whose cancers were AR+.^9^ Interestingly, one of the clear cell cases harbored a *CDKN2A (P16INK4A)* gene mutation; several studies have revealed mutations in this gene in a proportion of breast carcinomas [reviewed in^10^]. The discovery of genetic alterations of *CDKN2A* as well as other cell cycle regulators in breast cancers has led to the approval of CDK4/6 inhibitors (palbociclib) for the treatment of ER+/HER2‐ advanced/metastatic breast carcinomas.^10^, ^11^ In recent years, immunotherapy based on immune checkpoint inhibitors (against PD‐1/PD‐L1) has dramatically improved the treatment options and outcomes of several cancers including TNBC. The selection of patients for these drugs is based on several predictive biomarkers (I‐O biomarkers) including PD‐L1 expression (on cancer or immune cells) and TMB and MSI status. The status of I‐O biomarkers in clear cell carcinomas indicates a limited therapeutic benefit of immune checkpoint inhibitors (against PD‐1/PD‐L1). The presence of a *PTEN* mutation in one of the PD‐L1+ cases may suggest resistance to immune checkpoint inhibitors.^12^, ^13^ Nevertheless, finding immune cell PD‐L1 expression in a subset of clear cell carcinomas warrants further investigations given the approved treatment for TNBC with atezolizumab is solely based on immune cell expression of PD‐L1 (source: FDA, https://www.fda.gov/drugs/drug‐approvals‐and‐databases/fda‐approves‐atezolizumab‐pd‐l1‐positive‐unresectable‐locally‐advanced‐or‐metastatic‐triple‐negative, accessed on March 2, 2020). MSI and TMB status in clear cell carcinomas of the breast is similar to that in IBC‐NST.^14^, ^15^

In conclusion, clear cell carcinomas of the breast have limited targeted therapy options, but comprehensive molecular profiling may guide single or combined targeted treatments in selected cases.

## CONFLICT OF INTEREST

Jeffrey Swensen, Rebecca Feldman, Elma Contreras, and Elena Florento are employees of Caris Life Sciences. The other authors declare no conflict of interest.

## Supporting information

Table S1
