# Supplementary material for: Novel targetable biomarkers in clear cell carcinoma of the breast uncovered by molecular profiling: A study of nine cases
Source: Breast J. 2020 Apr 11;26(9):1781–3. doi: 10.1111/tbj.13842 (PMC7586831; doi:10.1111/tbj.13842)
Supplement: Supplementary file 1 — Table S1 [file TBJ-26-1781-s001.docx]

**Immunohistochemistry (IHC)**

| **Antibody** | **Clone (Manufacturer)** | **Threshold for positivity*** |
| --- | --- | --- |
| Estrogen receptor (ER) | SP1 (Ventana) | ≥1% cancer cells |
| Progesterone receptor (PR) | 1E2 (Ventana) | ≥1% cancer cells |
| Androgen receptor (AR) | AR27 (Leica Biosystems) | ≥10% cancer cells |
| ARv7 | EPR15656 (Abcam) | Any positivity in cancer cells |
| Her-2/neu | 4B5 (Ventana) | score 3+ by IHC or *HER2/CEP17* ratio ≥2.0 by ISH |
| PD-L1 | SP142 (Ventana) | ≥ 1% of the tumor area |
| pNTRK | EPR17341 (Abcam) | ≥1% cancer cells |
| PTEN | 6H2.1 (Dako Agilent) | Any positivity in cancer cells |
| MLH1 | M1 (Ventana) | Any positivity in cancer cells |
| MSH2 | G219-1129 (Ventana) | Any positivity in cancer cells |
| MSH6 | 44 (Cell Marque) | Any positivity in cancer cells |
| PMS2 | EPR3947 (Cell Marque) | Any positivity in cancer cells |

**Supplemental Table 1**. The list of antibodies that were used for the profiling of clear cell carcinoma of the breast.

*References:

1. Gatalica Z, Vranic S, Kruslin B, Poorman K, Stafford P, Kacerovska D, et al. Comparison of the biomarkers for targeted therapies in primary extra-mammary and mammary Paget's disease. Cancer Med. 2020;9(4):1441-50.
2. Vranic S, Senarathne W, Stafford P, Poorman K, Pockaj B, Gatalica Z. Biomarkers of targeted therapy and immuno-oncology in cancers metastatic to the breast. Appl Immunohistochem Mol Morphol 2019 Sep 11 Epub Ahead of Print, doi: 10.1097/PAI.0000000000000808
3. Gatalica Z, Xiu J, Swensen J, Maney T, Vranic S. Molecular characterization of cancers with NTRK gene fusions. Mod Pathol 2019;32:147-53.
4. Vranic S, Palazzo J, Sanati S, Florento E, Contreras E, Xiu J, Swensen J, Gatalica Z. Novel predictive biomarkers of targeted treatment for patients with neuroendocrine carcinoma of the breast. Clin Breast Cancer 2019;19:131-6.
5. Vranic S, Stafford P, Palazzo J, Skenderi F, Swensen J, Xiu J, Spetzler D, Gatalica Z. Molecular profiling of the metaplastic spindle cell carcinoma of the breast reveals potentially targetable biomarkers. Clinical Breast Cancer 2020 Feb 27 Epub Ahead Print, <https://doi.org/10.1016/j.clbc.2020.02.008>
6. Hammond ME, Hayes DF, Dowsett M, et al. American Society of Clinical Oncology/College of American Pathologists guideline recommendations for immunohistochemical testing of estrogen and progesterone receptors in breast cancer (unabridged version). Arch Pathol Lab Med 2010;134:e48-72.
7. Wolff AC, Hammond MEH, Allison KH, et al. Human Epidermal Growth Factor Receptor 2 Testing in Breast Cancer: American Society of Clinical Oncology/College of American Pathologists Clinical Practice Guideline Focused Update. J Clin Oncol 2018;36:2105-2122.
8. Schmid P, Adams S, Rugo HS, et al. Atezolizumab and Nab-Paclitaxel in Advanced Triple-Negative Breast Cancer. N Engl J Med 2018;379:2108-2121.
9. <https://www.accessdata.fda.gov/scripts/cdrh/cfdocs/cfpma/pma.cfm?id=P160002S009>
